# The Feasibility of Computer-Based Prism Adaptation to Ameliorate Neglect in Sub-Acute Stroke Patients Admitted to a Rehabilitation Center

**DOI:** 10.3389/fnhum.2013.00353

**Published:** 2013-07-24

**Authors:** Miranda Smit, Stefan Van der Stigchel, Johanna M. A. Visser-Meily, Mirjam Kouwenhoven, Anja L. H. Eijsackers, Tanja C. W. Nijboer

**Affiliations:** ^1^Rudolf Magnus Institute of Neuroscience, Center of Excellence for Rehabilitation Medicine, University Medical Center Utrecht and De Hoogstraat, Utrecht, Netherlands; ^2^Department of Experimental Psychology, Utrecht University, Utrecht, Netherlands; ^3^Rehabilitation Centre De Hoogstraat, Utrecht, Netherlands; ^4^Department of Neurology, University Medical Center Utrecht, Utrecht, Netherlands

**Keywords:** neglect, stroke, feasibility, efficacy, computer-based assessment, computer-based prism adaptation

## Abstract

**Introduction:** There is wide interest in transferring paper-and-pencil tests to a computer-based setting, resulting in more precise recording of performance. Here, we investigated the feasibility of computer-based testing and computer-based prism adaptation (PA) to ameliorate neglect in sub-acute stroke patients admitted to a rehabilitation center.

**Methods:** Thirty-three neglect patients were included. PA was performed with a pair of goggles with wide-field point-to-point prismatic lenses inducing an ipsilesional optical shift of 10°. A variety of digitalized neuropsychological tests were performed using an interactive tablet immediately before and after PA.

**Results:** All 33 patients [mean age 60.36 (SD 13.30)], [mean days post-stroke 63.73 (SD 37.74)] were able to work with the tablet and to understand, perform, and complete the digitalized tests within the proposed time-frame, indicating that there is feasibility of computer-based assessment in this stage post-stroke. Analyses of the efficacy of PA indicated no significant change on any of the outcome measures, except time.

**Discussion:** In conclusion, there is feasibility of computer-based testing in such an early stage, which makes the computer-based setting a promising technique for evaluating more ecologically valid tasks. Secondly, the computer-based PA can be considered as a reliable procedure. We can conclude from our analysis, addressing the efficacy of PA, that the effectiveness of single session PA may not be sufficient to produce short-term effects on our static tasks. Further studies, however, need to be done to evaluate the computer-based efficacy with more ecologically valid assessments in an intensive double-blind, sham-controlled multiple PA treatment design.

## Introduction

One of the major recent advances in neuropsychology is the use of computers during both screening and rehabilitation. There is wide interest in transferring paper-and-pencil tests to a computer-based setting (Schatz and Browndyke, 2002), resulting in a more detailed and precise recording of performance during screening and training (Rabuffetti et al., 2002; Chiba et al., 2006; Tsirlin et al., 2009) as well as enhanced consistency in testing across settings, making comparisons across patients more valid. Consequently, as the prism adaptation (PA) procedure has a fairly easy and repetitive design (see below), it is a good candidate for computer-based rehabilitation. Here, we investigate the feasibility of computer-based assessment and PA to ameliorate neglect in sub-acute stroke patients admitted to a rehabilitation center.

Neglect is a disabling disorder that frequently occurs after right hemisphere stroke (Bowen et al., 1999; Ringman et al., 2004). It refers to the failure to report, respond, or orient to stimuli on the contralesional side of space or body that cannot be accounted for by primary sensory or motor deficits (Halligan and Marshall, 1991; Robertson, 1999). Neglect is associated with poor functional recovery (Cherney et al., 2001; Jehkonen et al., 2006). Farne et al. (2004) found that whereas 43% of neglect patients demonstrated spontaneous recovery in the first 2 weeks, only 9% recovers completely. These findings concur with a recent study where patients were assessed several times during 1 year post-stroke (Nijboer et al., in press). In this study, spontaneous recovery of neglect appears to occur mainly during the first 12–14 weeks after stroke (Nijboer et al., in press) even though approximately 40% of the neglect patients do not fully recover and still show neglect on neuropsychological tests a year after stroke (Karnath et al., 2011; Rengachary et al., 2011; Nijboer et al., in press). Development of effective treatment techniques is therefore an important aim in neglect research, especially in the sub-acute phase, as the brain is primed to neurological recovery in the first 3 months post-stroke (Kwakkel et al., 2004; Murphy and Corbett, 2009).

One of the most widely investigated techniques to ameliorate neglect is PA (Rossetti et al., 1998; for overview, see Newport and Schenk, 2012). PA, originally proposed by Rossetti et al. (1998), is a promising experimental technique with strong therapeutic potential (Kerkhoff and Schenk, 2012). PA induces a proprioceptive shift in space by repetitive pointing to visual targets, resulting in a recalibration of the egocentric coordinate system. In other words, it creates a pointing bias in the opposite direction after prism removal and a contralesional shift in subjective body midline (Heilman et al., 1983; Saj and Vuilleumier, 2007). Positive effects of PA have been reported across many visuo-manual tasks in patients in the chronic phase, such as bisecting lines, line crossing, copy drawing (Saevarsson et al., 2010; Striemer and Danckert, 2010; Sarri et al., 2011), but also in more non-manual tasks, such as picture scanning, object-naming tasks and reading tasks (words and non-words) (Farne et al., 2002), and daily situations, such as wheelchair navigation (Rossetti et al., 1999; Watanabe and Amimoto, 2010) and postural control (Tilikete et al., 2001). The beneficial effects of PA have been reported to last 2 h (Rossetti et al., 1998) up to 1 week (Pisella et al., 2002; Dijkerman et al., 2004), and even up to 2 years (Nijboer et al., 2011).

Notwithstanding these promising results, evidence on feasibility and secondarily the efficacy of PA in the sub-acute stroke stage in a rehabilitation setting is relatively scarce. Nonetheless, it is important to identify an optimal or “critical period” for the optimal treatment response, keeping in mind that neurological recovery takes place in the first 3 months post-stroke (Kwakkel et al., 2004; Murphy and Corbett, 2009; Nijboer et al., in press). There are only a few randomized control trials that assessed the efficacy of PA exclusively in sub-acute (range 2–86 days) stroke patients (Nys et al., 2008; Turton et al., 2010; Mizuno et al., 2011). The effectiveness of PA in this stage remains equivocal, however. Whereas Mizuno et al. (2011) performed an intensive 2-week PA treatment and found improvement on the conventional Behavioral Inattention Test (BIT) and on a functional independence measure, Turton et al. (2010) did not find such an effect. The lack of efficacy in the latter study might be attributable to the use of 6° goggles, which is a lesser degree of lateral displacement than that used in other studies (Barrett et al., 2012). Nys et al. (2008) only found short-term superiority in performance on the BIT, compared to placebo treatment. Clearly, the effectiveness of PA in the sub-acute stage post stroke needs further research.

To date, we do not know whether testing in general and computer-based assessment is too difficult or too time-consuming in this stage of syndrome since many previous studies were performed in the chronic stage. Therefore, our aim is to investigate the feasibility of computer-based assessment in a sub-acute stage post-stroke. Secondly, we combined the widely used PA procedure with a computer-based setting in order to investigate the feasibility of computer-based treatment (PA). Importantly, it is unknown whether a prismatic after-effect can be obtained with computer-based treatment. Furthermore, we gain insight in the adaptation procedure by means of more detailed and precise recordings of the pointing movements during the adaptation procedure as well as the magnitude of the after-effect. Lastly, we want to investigate the efficacy of a single session of computer-based PA on neuropsychological digitalized tests.

## Methods

### Participants

In this study 33 stroke patients [mean age 60.36 (SD 13.30); mean days post-stroke 63.73 (SD 37.74)] with neglect (31 left visuospatial neglect) were included (see Table 1 for patient characteristics). All patients were admitted to rehabilitation center de Hoogstraat. Patients were included when they met the following criteria: (1) a brain lesion as revealed by CT or MRI; (2) presence of spatial neglect as assessed with a short screening including the Object Cancelation and Letter Cancelation (see Inclusion Based on Short Neuropsychological Screening below in this paragraph); (3) aged between 18 and 80 years; (4) able to understand and carry out the test instructions; (5) written or verbal informed consent and sufficient motivation to participate.

**Table 1:** **Demographical and stroke characteristics of the included patients**.

Clinical variables	Included patients (SD)
Group size	33
Age (years)	60 (13.30)
Gender (male)	57.58%
**Stroke characteristics**	
Days post-stroke	63.73 (37.74)
Hemisphere of stroke (*R*)	90.91%
Unilateral	96.97%
Type of stroke
Cortical ischemia	63.64%
Subcortical ischemia	3.03%
Intracerebral hemorrhage	30.30%
Other*	3.03%
Barthel index (*n* = 28)	12.07 (5.77)
Motricity index arm (*n* = 23)	57.43 (41.38)
Motricity index leg (*n* = 23)	67.43 (36.75)
MMSE (*n* = 25)	25.54 (4.29)
Hemianopia	39.39%

MMSE, mini mental state exam; *ischemia due to acute disseminated encephalomyelitis.

All patients received multidisciplinary standard stroke care and treatment and participating in the study did not interfere with daily routines. Additionally, all patients received visual scan training, which was the current intervention used for rehabilitation of neglect in this rehabilitation center.

#### Inclusion based on short neuropsychological screening

The short screening that took place prior to the inclusion of the present study was part of the standard stroke care. At this level, severity of neglect was evaluated on the bases of the standard outcome measures of the Object Cancelation and the Letter Cancelation (number of omissions). The average number of omissions was 7.21 (SD = 8.39) for the Object Cancelation and 7.11 (SD = 5.80) for the Letter Cancelation. It should be noted that only 15 patients of our total sample performed the Letter Cancelation in the screening. When evaluating the level of lateralized impairment, we calculated the asymmetry in the number of omitted items. For the Object Cancelation, 27 patients (out of 33) showed an asymmetry in omitted items in the range from 0 to 10 (*M* = 2.44; SD = 2.39) and 6 patients had an asymmetry score between 15 and 30 (*M* = 19.33; SD 3.78). For the Letter Cancelation test (LC), 16 (out of 18) patients displayed an lateralized deficit between 0 and 10 (*M* = 3.63; SD 3.07) and 2 patients had an asymmetry score between 10 and 15 (*M* = 14.00; SD 1.41).

### Apparatus

Both the PA procedure and the neuropsychological tests were done using a 22-inch interactive WACOM (PL2200) tablet screen (1920 × 1080), with a screen size of 477.64 mm × 268.11 mm. The tablet includes a widescreen display (luminance: 200 cd/m^2^) and full HD resolution (0.01 mm/point) and has a screen refresh rate of 5 ms. The display offers a large working area and provides good spatial (±0.01 mm/point) and temporal resolution (133 points/s, max).

Patients had to respond to stimuli by drawing on or pointing at the screen with a digital stylus. We used an electromagnetic resonance method to record patients’ performance of the stylus. DiagnoseIS (developed by Metrisquare, Netherlands) was used to program the neglect screening tests (e.g., Cancelation tests). The tablet was driven by a laptop in order to monitor stimuli and patients performance on the experimenter’s laptop. During performance the tablet screen was oriented horizontally and slightly tilted (18°) with an adjustable stand.

### Digitalized prism adaptation procedure

The PA procedure was adapted from Rossetti et al. (1998) and was performed with a pair of goggles fitted with wide-field point-to-point prismatic lenses, inducing a rightward optical shift of 10°.

The distance between the visual stimuli and the body midline was approximately 65 cm. Patients were presented with three visual targets (red, yellow, blue) on a horizontal axis. The left and right visual targets were both 11.5 cm away from a central visual stimulus. Exposure consisted of 100 fast repetitive pointing movements. Half of the pointing movements were made to the left visual target, the other half were made to the right visual target. Patients were occasionally instructed to point to the central visual stimulus when pointing appeared to become a routine. These additional pointing movements prevented automatic pointing in a sequence of motor acts to either the left or the right target. When patients experienced difficulties in distinguishing between left and right, the color of the visual stimulus was used. Whenever the patient touched the tablet screen with the digital stylus, *x* and *y* coordinates and timing data were recorded. Error reduction was achieved when patients hit the target. Patients pointing performance was only and immediately presented at the laptop of the experimenter, allowing the experimenter to monitor the accuracy of the pointing movements online.

After the adaptation phase (e.g., repetitive pointing), prisms were withdrawn and the after-effect was measured. Conventionally, the strength of the adaptation can be obtained by measuring the spatial deviation from a target stimulus. During the repetitive pointing movements (the adaptation phase), visuo-motor corrections toward the contralateral side in order to point to the target as accurate as possible, are executed. Thus, when prisms are removed, the spatial deviation will be in the opposite direction of the visual displacement imposed by the prism glasses, a phenomenon known as the after-effect. In our sample we used the central target to measure the after-effect. Here, after prism removal, patients were instructed to look carefully at the central visual target. After a few seconds they were instructed to point with the digital stylus at the central target, with eyes closed to prevent online adjustments. Again, patients did not get feedback about the landing position of the digital stylus, which was only shown at the experimenter’s laptop. For after-effects, the mean error displacement from the central stimulus was calculated and should have been at least 3 cm, otherwise the PA procedure was continued.

### Stimuli, tests, and procedure

All measurements were conducted in a sound-attenuated room. Patients were seated as comfortable as possible, in upright position in front of the tablet. All tests were done using the tablet. These tests were done prior and immediately after PA in the same order if possible. The whole test procedure lasted for an hour and patients were allowed to have a small break when needed prior or after the PA procedure.

In an *Object Cancelation* test (OC), patients were presented with 54 targets, and 75 distractors. Patients had to cross out all the targets. Patients were given feedback; they could see their own performance; e.g., see the stripe of the digital stylus through the canceled items. Outcome measurements were the total number of omissions, total time for test completion, search time in the ipsilesional and contralesional field and the horizontal and vertical Center of Cancelation (CoC). The CoC is an indicative measure of severity of neglect, since it obtains information of both the number of omissions and the location of canceled items. Generally, a positive CoC-score (+) indicates that the mean horizontal location of the canceled items is at the right side of the stimulus sheet, e.g., indicating lateralized deficits on the far left and vice versa. A CoC-score toward zero means a more symmetrical spatial error distribution. Calculations for the CoC were adapted from Rorden and Karnath (2010). Additionally, the same method was applied for the number and spatial distribution of perseverations.

The *Letter Cancelation* test (LC) consisted of 5 rows of 34 random letters (170 letters in total). Patients were instructed to cancel the target letters, which were randomly placed between the distractor letters. Outcome measures were the total number of omissions, total time for test completion, search time in the ipsilesional and contralesional field and the horizontal CoC (Rorden and Karnath, 2010).

In a *Line bisection* test (LB), patients were presented with three horizontal lines (31 cm in length and 1 mm in width). The lines were outlined in a staircase fashion and patients had to indicate (upper to low) the true center of each line. This test was performed twice. Outcome measurement was the total deviation of the true midpoint for each line and total time for test completion.

### Analyses

In the first part of the result section qualitative information about the feasibility of computer-based testing and computer-based treatment will be addressed. Moreover, reasons of exclusion will be specified.

In the second part, analyses of the efficacy of PA will be discussed. Regarding the cancelation tests; 27 and 26 patients were included in the OC and LC, respectively (see below for reason of exclusion). For both tasks paired sample *t*-tests were performed between pre- and post-test for total number of omissions, total time for test completion, and the search-times on both the ipsilesional and contralesional side. Moreover, the mean CoC were calculated for both the cancelation tasks. The mean Center of Perseveration was only calculated for the OC, due to a small amount of perseverations in the LC. Paired samples *t*-tests were performed between the pre-horizontal and vertical CoC/CoP and the post-horizontal and vertical CoC/CoP.

For the LB, 27 patients were included (see below for reason of exclusion). Paired samples *t*-tests were performed between pre-session and post-session for the mean deviation of the true center (i.e., mean line 1^a^ pre-test and line 1^b^ pre-test versus mean line 1^a^ post-test and line 1^b^ post-test, likewise for line 2 and 3) and total time for test completion. For all tests, since we had both left and right neglect patients in our sample, we made “contralesional” and “ipsilesional” classifications in order to consider them as one group. A two-tailed significance level of 0.05 was used. Results of the efficacy of PA on the digitalized tests are outlined in Table 2.

**Table 2:** **Mean results of the digitalized pre-test and post-test**.

Test	*N*	Outcome measure	Mean pre-test	Mean post-test	Statistics
OC	27	Omissions total	4.48 (6.44)	5.37 (6.80)	*t*(26) = −1.464, *p* = 0.155
	27	CoC-*x*	0.04 (0.13)	0.05 (0.13)	*t*(26) = −0.417, *p* = 0.680
		CoC-*y*	0.00 (0.02)	0.00 (0.03)	*t*(26) = −0.910, *p* = 0.371
	27	Perseverations total	7.04 (10.87)	6.52 (11.83)	*t*(26) = 0.517, *p* = 0.610
	27	CoP-*x*	0.08 (0.30)	0.06 (0.41)	*t*(26) = 0.265, *p* = 0.793
		CoP-*y*	0.08 (0.29)	0.06 (0.36)	*t*(26) = 0.232, *p* = 0.818
	25^1^	Total time in sec	99.16 (40.85)	84.00 (37.21)	*t*(24) = 3.318, *p* = 0.003**
		Time contralesional	32.40 (18.38)	25.80 (16.05)	*t*(24) = 3.234, *p* = 0.004**
		Time ipsilesional	33.70 (16.02)	26.80 (12.23)	*t*(24) = 4.082, *p* < 0.0001***
LC	26	Omissions total	6.23 (7.00)	5.00 (5.69)	*t*(25) = 1.786, *p* = 0.086
	26	CoC-*x*	0.06 (0.16)	0.04 (0.14)	*t*(25) = 1.126, *p* = 0.271
		CoC-*y*	0.00 (0.04)	0.00 (0.03)	*t*(25) = 0.088, *p* = 0.931
	25^1^	Total time in sec	106.14 (49.63)	93.09 (31.73)	*t*(24) = 2.394, *p* = 0.025*
		Time contralesional	25.52 (11.78)	22.85 (11.28)	*t*(24) = 2.357, *p* = 0.027*
		Time ipsilesional	28.54 (10.03)	27.93 (9.89)	*t*(24) = 0.422, *p* = 0.677
LB (mm)	27	Deviation line 1^a^1^b^	−1.97 (18.30)	−1.81 (12.82)	*t*(26) = −0.061, *p* = 0.952
		Deviation line 2^a^2^b^	−9.30 (21.68)	−8.77 (19.34)	*t*(26) = −0.268, *p* = 0.791
		Deviation line 3^a^3^b^	−17.75 (28.13)	−23.78 (29.61)	*t*(26) = 1.672, *p* = 0.106
		Total time	10.19 (6.42)	7.94 (4.34)	*t*(26) = 2.458, *p* = 0.021*

Standard deviations are given in parenthesis. OC, Object Cancelation; LC, Letter Cancelation; CoC, Center of Cancelation; CoP, center of perseveration; LB, Line Bisection, deviation score in millimeters, − deviation to the right, + deviation to the left; *significant<0.05, **<0.005, ***<0.0001. ^1^Specific timing data was due to a synchronization error not stored for two patients in the OC, and one patient in the LC.

## Results

### Feasibility of computer-based testing

First, all 33 patients were capable to respond to stimuli by drawing, canceling on, or pointing at the screen with a digital stylus, indicating that computer-based testing was feasible. Second, considering the overall feasibility of performing tests in such an early stage post-stroke we observed that, all but one patient, performed the pre- and post OC and LB; that particular patient did not complete the post-test (as well for the LC) due to emotional factors. For the LC, 31 patients performed the pre- and post-test. One patient did not perform the pre- and post LC due to a language barrier and illiteracy. Third, patients were able to complete test performance within the proposed time-frame. The total duration of performing all digitalized tests, pre and post, was approximately 6.7 min, which indicates that after the verbal instruction was given, patients worked continuously on the digitalized tasks, meaning that test instructions were understood quickly. The total duration of the PA (first pointing movement till after-effect) was, on average, 8.4 min.

### Feasibility of computer-based PA in sub-acute stroke patients

Generally, patients were able to perform the PA procedure, e.g., pointing at the screen with a digital stylus, on a computer. However, for some patients the PA procedure (repetitive pointing movements) was sometimes strenuous. Four patients experienced difficulties distinguishing left from right. Two out of four of these patients preferred color naming over left-right responses. Additionally, one patient experienced a headache while pointing. This became less when the adaptation procedure proceeded. Due to repetitive pointing one patient experienced exhaustion of the right arm. For another patient, working with the digital stylus became difficult as a result of rheumatic problems.

Additionally, a Pearson correlation coefficient was performed in order to assess whether a relationship existed between neglect severity at baseline and the magnitude of the error displacement. Neglect severity was assessed with the “asymmetry score” of the OC from the neuropsychological screening, see participant section. However, Pearson correlation coefficient revealed no significant relationship between the magnitude of the error displacement and the asymmetry score in the OC, *r* = 0.132, *n* = 33, *p* = 0.463. This indicates that neglect severity was not associated with the level of adaptation.

**Figure 1:**
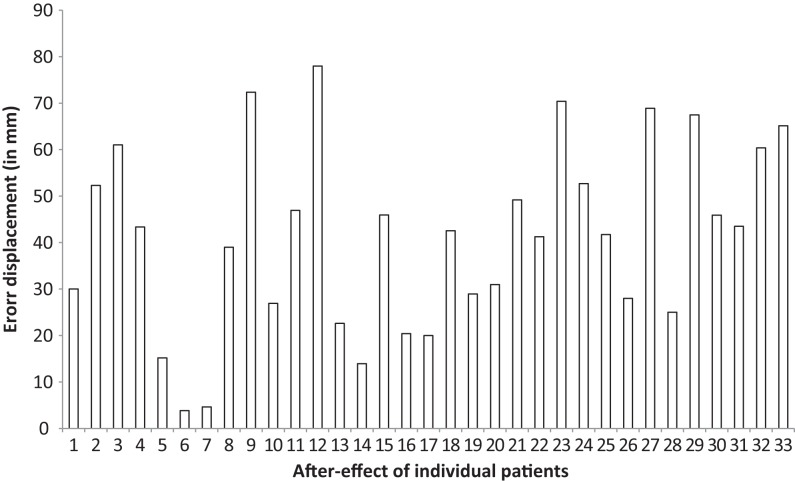
**The error displacement (after-effect after the adaptation phase) in millimeters from the central target for each individual patient**.

To recall, the strength of the adaptation was obtained by measuring the spatial deviation (in cm) from the central stimulus, which is called the after-effect phenomenon (see Digitalized Prism Adaptation Procedure in Methods section). Regarding the magnitude of the after-effect, the mean error displacement from the center target of all the 33 patients was 4.12 cm (SD 2.00) with a minimum displacement of 0.38 and a maximum displacement of 7.24, indicating that most, but not all patients, adapted well to the prism procedure (see Figure 1). For five patients the adaptation procedure was continued, and after a second adaptation phase, these patients still showed a minor error displacement (*M* = 1.26; SD = 0.81). These patients were not included in the analyses on the efficacy of the digitalized neuropsychological tests (see below). One patient whom had also a small after-effect was not secondly adapted due to emotional factors (see above).

### Excluded patients

In sum, six patients were excluded from the overall analyses in the LB and OC. For the LC, seven patients were excluded.

### Recordings of the pointing movements during the adaptation procedure

**Figure 2:**
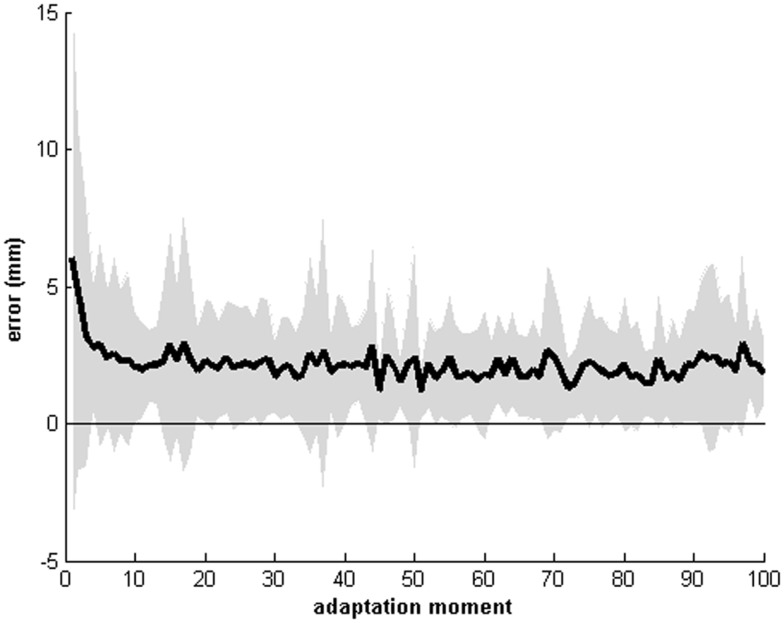
**Mean recordings of the pointing movements of all 33 patients for the first 100 pointing movements**. The horizontal axis displays the moment of pointing (0 till 100), the vertical axis displays the error displacement of either the right, left, or central target. Shaded area indicates the mean standard deviation. Note that the absolute center of each target (*x*, *y* coordinate) was used as the referent.

**Figure 3:**
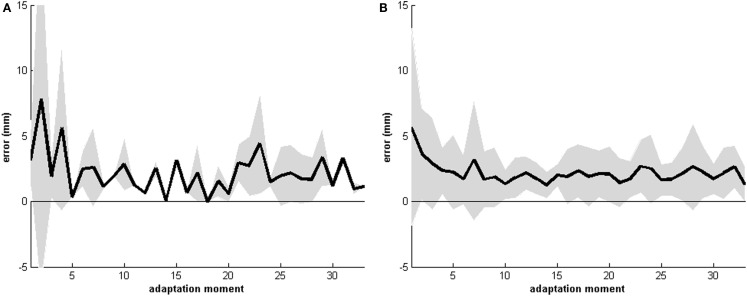
**Mean recordings of the pointing movements of all 33 patients for 30 pointing movements for either the “right” (A) and the “left” (B) target**. The horizontal axis displays the moment of pointing (0 till 30), the vertical axis displays the error displacement from the right **(A)** or the left **(B)** target. Shaded area indicates the mean standard deviation. Note that the absolute center of the target (*x*, *y* coordinate) was used as the referent.

Recordings of the pointing movements revealed that the error displacement was the largest at the first five pointing movements (see Figures 2 and 3). Thereafter the error displacement became relatively stable and patients become fairly accurate in pointing to either the left, right, or central target. This indicates that the process of recalibrating the new egocentric coordinate system sets in rapidly. Note that the absolute center of each target was used as a referent and not the whole target. In this regard, patients could have hit the target (i.e., error reduction was achieved), but not the true center (*x*, *y* coordinate) of that target.

### Efficacy of a single session of computer-based PA

Analyses of the efficacy of PA did not reveal significant changes in deviation from the actual center on the line bisection nor in number of omissions on the cancelation tests. Additionally, no significant shift in the location of the omissions and perseverations was found (CoC and CoP outcome measures). The total time taken to complete the test improved significantly after PA for the OC, LC, as well as the LB. Results of the digitalized tests are outlined in Table 2.

Regarding time for test completion, all tests were performed significantly faster (total time) after PA, see Table 2. Additionally, when addressing the search-times (Table 2) on either the ipsilesional as well as the contralesional side of the stimulus-sheets presented on the tablet, patients performed significantly faster post PA in the OC. For the LC this was only faster on the contralesional side post PA. Search-times were based on the search-times within one field, and could reflect either searching a target but also recursively exploring one side due to perseveratory behavior.

## General Discussion

The aim of the current study was to (1) investigate the feasibility of computer-based testing (2) and computer-based treatment in sub-acute stroke patients; (3) gain insight in the adaptation procedure by means of more detailed and precise recordings of pointing movements; and (4) investigate efficacy of a single session of computer-based PA on neuropsychological digitalized tests. Regarding the feasibility of computer-based neuropsychological assessment, all of the included patients were able to work with the tablet and to understand and perform the digitalized tests within the proposed time-frame. This indicates that there is feasibility of computer-based testing in an early stage post-stroke. Although, using this type of tablet and stylus was indeed feasible in our sample, it should be noted that our sample was relatively young and suffered from relatively mild neglect on neuropsychological neglect screening tests. Moreover, our sample rehabilitated with the intention to reintegrate in society as soon as possible. One might assume that these patients display fewer problems (both physically and mentally) than long stay stroke patients whom live in nursing homes. Our sample may thus not be representative for the overall stroke population. In this regard, a finger activated tablet might be a more appropriate solution in chronic patients or during bedside testing.

Not only can computer-based testing improve the traditional paper-and-pencil assessment of neglect by means of more precise and detailed recordings (Rabuffetti et al., 2002; Chiba et al., 2006; Tsirlin et al., 2009), it also holds opportunities in developing more ecologically valid tests. For instance, daily situations are far more dynamic and require fast responses in order to avoid obstacles in complex environments. We suggest that dynamic tests (e.g., ecologically based) increases the attentional load similar to daily life and will be more sensitive in detecting the “real level of neglect” (Tsirlin et al., 2009). This is impossible with the traditional paper-and-pencil tests.

In addition, the computer-based treatment can be considered as a reliable tool in performing PA, since most patients adapted well to the procedure, as quantified with the magnitude of the after-effects. These after-effects were comparable with our traditional (paper and pencil) PA after-effects (Nijboer et al., 2008, 2010, 2011; Bultitude et al., 2013). Moreover, detailed recordings of the pointing movements revealed that, on average, the error displacement was the largest for the first five pointing movements. Thereafter the error displacement was relatively stable and patients became fairly accurate in pointing to either the left, right, or central target. This implies that the process of recalibrating a new egocentric coordinate system sets in rapidly. Combining computer-based treatment with computer-based ecologically valid assessments in the sub-acute stage seems like a promising thought in identifying a “critical period” for the optimal treatment response with more detailed measures, especially when neurological recovery takes place in the first 3 months post-stroke (Kwakkel et al., 2004; Murphy and Corbett, 2009; Nijboer et al., in press).

Analyses of the efficacy of PA indicated no significant change on any of the outcome measures, except time. However, our sample as a group showed only mild visual neglect at baseline in especially the cancelation tasks. In this regard, there was less room for further improvement. Second, it is likely that concurrent compensation training already changed the scanning strategy in these neglect patients and that one session of PA does not further enhance attentional processing. In addition, since our tests were statically presented at the tablet till task-completion, patients could apply their in-hospital learned cognitive strategy (top-down scanning strategies toward contralesional stimuli), which could have masked their real level of neglect. It would have been interesting to investigate differences in feasibility (pointing movements) and efficacy in both left- and right-sided neglect. The sample size of especially the group of right-sided neglect patients (2) was too small, however, to statistically compare the efficacy of single session PA between those two groups.

Moreover, our design was not fit to fully evaluate effects of PA. We did not use a control-group to counteract learning- and/or motivational-effects. Although we lacked an effect of PA on the digitalized tasks, we do not know whether PA had no effect at all. Subtle treatment effects could be overruled by fatigue at the end of the test-session, since post-stroke fatigue is common (De Groot et al., 2003; Lerdal et al., 2009). Furthermore, one session may be insufficient to produce long-term and even short-term effects, which is in line with the conclusion in a recent review (Schenk and Karnath, 2012). However, generalizable and stable improvement of PA was found when using an intensive treatment program with multiple sessions (10 or more) of PA (Frassinetti et al., 2002; Serino et al., 2009; Mizuno et al., 2011). In addition, we do not know whether a computer-based setting influenced test performance either positively or negatively. It is possible that patients were more alert at the start of the novel test-situation (with a tablet) and became less alert when the computer-based setting became more familiar. In order to disentangle treatment effects from motivational effects and to control for a computer-based setting, a sham-controlled design is necessary.

In conclusion, there is feasibility of computer-based testing in such an early stage, which makes the computer-based setting a promising technique for evaluating more ecologically valid tasks. Secondly, the computer-based PA can be considered as a reliable procedure. We can conclude from our analysis, addressing the efficacy of PA, that the effectiveness of single session PA may not be sufficient to produce short-term effects on our static tasks. Further studies need to be done to evaluate the computer-based efficacy with more ecologically valid assessments in an intensive double-blind, sham-controlled multiple PA treatment design.

## Conflict of Interest Statement

The authors declare that the research was conducted in the absence of any commercial or financial relationships that could be construed as a potential conflict of interest.
